# COVID-19 and Its Impact on Healthcare Workers: Understanding Stigma, Stress, and Quality of Life

**DOI:** 10.7759/cureus.37846

**Published:** 2023-04-19

**Authors:** Abdullah Alhouri, Muntaser Abu Shokor, Khaled Marwa, Alaa Sharabi, Douaa Mohammad Nazir Arrouk, Fatima N Al Houri, Hasan Al Houri

**Affiliations:** 1 Department of Internal Medicine, Royal Berkshire Hospital, Reading, GBR; 2 Department of Orthopaedics, Specialty Hospital, Amman, JOR; 3 Department of General Medicine, Al Safa Medical Complex, Hail, SAU; 4 Department of General Medicine, Somerian Health, Abu Dhabi, ARE; 5 Applied Statistics, Department of Quantitative Methods, Damascus University, Damascus, SYR; 6 College of Medicine, American University in the Emirates, Dubai, ARE; 7 Department of Internal Medicine, Syrian Private University, Damascus, SYR; 8 Department of Internal Medicine, Al-Assad University Hospital, Al-Mouwasat University Hospital, Damascus, SYR

**Keywords:** quality of life (qol), stigma in health care, covid 19, stress, : healthcare workers

## Abstract

Background: The coronavirus disease 2019 (COVID-19) pandemic has negatively impacted public health systems worldwide and created anxiety and stress among communities, resulting in the stigmatization of patients infected with the virus. Stigmatization of individuals who are sick or thought to be infected has a long history and can lead to discrimination and prejudice. This study aims to evaluate the prevalence of COVID-19-related stigma in Jordan, assess the relationship between stigma and the quality of life (QoL) in healthcare workers, and identify possible measures to decrease stressful events. Understanding the psychological effects of healthcare workers' jobs and reducing their burden is essential to improving medical outcomes and the QoL of patients.

Methodology: This cross-sectional study was conducted in three primary hospitals in Amman, Jordan, from July to December 2021. Healthcare workers were recruited through convenience sampling and completed a self-administered questionnaire, which included demographic information, a validated COVID-19 stigma questionnaire, work conditions during the pandemic, the Depression, Anxiety and Stress Scale - 21 Items (DASS-21) questionnaire to measure depression, anxiety, and stress, and the EUROHIS-QOL questionnaire to assess the QoL. Data were analyzed using descriptive and inferential statistics, including chi-square tests and post hoc analysis. The study was approved by the institutional review board, and participation was voluntary and confidential.

Results: The study was conducted among 683 healthcare workers in Jordan, with 77.7% based in the capital city, Amman. Most of the participants were between 18 and 30 years of age, and slightly more than half were female. The study found that 38.1% of healthcare workers would not take the COVID-19 vaccine once it became available. More than half (56%) reported experiencing stress, 61% reported anxiety, and 65% reported depression during the pandemic. Internal medicine specialties and frontline nurses reported the highest levels of stress, and healthcare workers with greater exposure to COVID-19 patients reported higher levels of anxiety and stress. Only 3% of participants reported experiencing stigmatization (p=0.043), with low-income participants reporting it more frequently. Stigmatization was significantly correlated with feelings of depression, anxiety, and stress (p<0.001).

Conclusion: Healthcare workers’ mental well-being has been affected negatively by the COVID-19 pandemic, resulting in depression, anxiety, and stress. Widespread mental surveillance for healthcare workers is crucial to protect healthcare workers from psychological issues and to improve the healthcare service provided to patients. Stigma among healthcare workers can be a major factor that may increase depression, anxiety, and stress.

## Introduction

Coronavirus disease 2019 (COVID-19) has become a global pandemic, infecting over 700 million confirmed cases and causing around 6.8 million deaths worldwide. This outbreak has created tremendous pressure on public health systems worldwide, particularly in developing countries. The pandemic's impact has negatively affected economies globally, making it a daunting challenge for governments to manage. Additionally, it has produced anxiety and stress among communities, stigmatizing patients infected with COVID-19 [1,2]. The World Health Organization (WHO) defines social stigma in health as the negative association between a person or group of people who share certain characteristics or a specific disease. During an outbreak, this may mean people are labeled, stereotyped, discriminated against, treated separately, and experience loss of status because of a perceived link with a disease.

Throughout history, societies have singled out or avoided groups with specific characteristics viewed as threatening or undesirable to others. Symptoms of illness, the perception that someone may be a silent carrier or even just being close to an ill patient, can lead to stigmatization. Previous infectious outbreaks of cholera, plague, and yellow fever led societies to marginalize individuals who were sick or thought to be ill or infected [3,4]. Between the 1920s and 1930s, the movements and homes of people infected with tuberculosis (TB) were monitored by health inspectors. Although these measures resulted in significant declines in TB cases then, they were unfortunately accompanied by noticeable discrimination and prejudice against those with TB, or the "lungers" as they were called [5]. While the fear of individuals with infectious diseases has facilitated disease avoidance among societies and raised personal hygiene, it has also increased discrimination and stigmatization of infected individuals, leading them to avoid seeking healthcare or following healthy behaviors. These barriers may result in ongoing transmission, difficulties in controlling infectious diseases during outbreaks, and more severe health problems [6].

It is essential to understand the key characteristics of healthcare workers, study the relationship between their work burden and quality of life (QoL) parameters, and recognize the psychological effects of their jobs. All of these could help optimize the usage of interventional and supportive measures and reduce the negative pressures on their lives. Reducing the burden on healthcare workers will improve their patients' medical outcomes and QoL [7,8]. Several causes stand behind this high level of stressful environments, such as night shifts, enormous workload, conflicts with colleagues or patients, and the severity of high illness [9]. Thus far, all of these factors can affect the social and mental health of healthcare workers; the addition of the COVID-19 pandemic has placed them in even more stressful circumstances with increased patient loads, unprecedented disruptions to everyday life, and a high risk of exposure, ultimately affecting the healthcare services delivered to patients [10].

Historically, stigmatizing healthcare workers has been a prevalent practice during epidemics. Nevertheless, despite the attention that healthcare professionals have received, there have been several reports of stigmatization among healthcare professionals worldwide. Adiukwu et al. have described COVID-19-related stigma that touched on the unfavorable vocabulary used to describe the coronavirus and those infected with it, widespread myths about COVID-19, behaviors associated with stigma, and attitudes toward particular ethnic groups [11-13]. This study aims to evaluate the prevalence of COVID-19-related stigma in Jordan, assess the relationship between stigma and the QoL in healthcare workers, and identify possible measures to decrease stressful events.

## Materials and methods

Study design and setting

This cross-sectional study was conducted in three primary hospitals (University of Jordan Hospital, Specialty Hospital, and Istiklal Hospital) that dealt with patients during the COVID-19 pandemic (both the first and second wave) in Amman, Jordan. Data was collected between July 1 and December 30, 2021 [14-16].

Ethical considerations

This study was approved by the Institutional Review Board (IRB: 103529) of Specialty Hospital. Participation was voluntary. The anonymity and confidentiality of the data were ensured by assigning an identification number to each participant restricted to the research team. The questionnaire contained detailed information about the study objectives, and returned questionnaires implied signed consent.

Sample selection

Healthcare workers were recruited through convenience sampling to participate in a self-administered questionnaire. Respondents eligible for the study were adult male and female healthcare workers of any age. The sample included physicians, dentists, nurses, pharmacists, and other medical technicians who signed consent forms before data collection.

Data collection

A detailed explanation of the study was presented to healthcare workers at participating hospitals and clinics. The researchers distributed the questionnaires to healthcare workers either directly or by email. Each questionnaire had a cover letter explaining the study, its aims, and how to complete and return the form. Participants had to sign consent forms, and the self-completed questionnaires were returned to the researchers directly.

Assessment of stigma, Depression, Anxiety, and Stress Scale (DASS), and QoL

The questionnaire included five main parts: The first part of the questionnaire collected the healthcare workers' demographic information, including their age, sex, place of residence, educational level, field of study, living status, number of people living with the respondent, and financial status.

The second part of the questionnaire consisted of a structured, validated, and published questionnaire used in the literature; we determine if healthcare workers experienced COVID-19-related stigma, regardless of whether they were in direct contact with COVID-19 patients or not [11]. The questionnaire focuses on two aspects of stigmatization, including perceived harmfulness and inferiority (seven items) and avoidance (four items) among healthcare workers. Responses were captured on a five-point Likert scale (never, rarely, sometimes, very often, and always), then converted to scores of 1, 2, 3, 4, and 5, respectively. The mean, standard deviations, and frequencies were calculated for descriptive statistics.

The third section of the questionnaire focused on participants' work conditions during the pandemic. The questions included whether they worked at a hospital or clinic and whether their neighbors, relatives, or coworkers knew about their employment at these facilities. Additionally, participants were asked if they worked with COVID-19 patients and if their contacts were mindful of this. The questions had yes or no responses and assessed the frequency and duration of exposure to COVID-19 patients. The frequency of working in health institutions was categorized into five options, from one to five days a week. The time of exposure was divided into two categories: close daily contact with protective equipment (PPE) and only occasional contact for a few minutes with PPE.

The fourth part of the questionnaire used the short version of the DASS (DASS-21) to measure depression, anxiety, and stress (25). The DASS-21 is a self-reported questionnaire comprising three seven-item scales designed to measure symptoms of depression, anxiety, and stress in the past week. The response categories for each scale range from 0 to 3, with higher scores indicating greater severity of symptoms. The total scores for each scale range from 0 to 21, and participants are categorized into four groups based on their full scores: minimal/no (0-4) symptoms, mild symptoms (5-9), moderate symptoms (10-14), or severe symptoms (15-21).

The fifth part of the questionnaire assessed the participants' QoL using the EUROHIS-QOL questionnaire, which included eight questions [13].

Statistical analysis

The Statistical Package for the Social Sciences (SPSS) software, version 25.0 (IBM Corp., Armonk, NY), was used for data analysis. The statistical significance level was set at (0.05). Descriptive statistics were used to examine the demographic characteristics of participants in the sample and analyze the distributions of responses to questionnaire items and each subscale.

Inferential statistics focuses on finding the correlation between different variables. Chi-square test and post hoc analysis (using the Scheffe test where appropriate) were used to analyze how stigma scores and DASS varied according to other demographic variables. The chi-square test was also used to assess the relationship between stigma scores and DASS. The normality of the data was checked to ensure that the assumptions of the statistical tests were met

## Results

Characteristics of study participants

This study was conducted among 683 healthcare workers in Jordan, with 531 (77.7%) based in Amman's capital city. Most participants (67.2%) were 18 to 30 years old, with slightly more than half (59.7%) female. Regarding housing, 76.3% of respondents reported living in their own houses, while 43.9% lived with five or more people in the same place. More than half of the participants said they had an excellent financial status, indicating that their salaries were sufficient to meet their basic needs with some luxury. Notably, 652 (95.5%) of the participants did not travel during the pandemic (Table 1).

**Table 1:** Characteristics of study participants personal protective equipment (PPE)

Variables (n=683)	Frequency	Percent
Age		
18-30	459	67.2
31-50	198	29.0
>=50	26	3.8
Gender		
Male	275	40.3
Female	408	59.7
Living condition		
University accommodation	17	2.5
House	521	76.3
Flat	145	21.2
Including yourself, how many people live in your household?		
Alone	37	5.4
1-2	106	15.5
3-4	240	35.1
≥5	300	43.9
Financial status		
Low	35	5.1
Medium	207	30.3
Good	352	51.5
Excellent	89	13.0
Have you traveled during the covid-19 period?		
No	652	95.5
Yes	31	4.5
Educational level		
Doctorate	21	3.1
Master degree	46	6.7
University degree	548	80.2
Institute degree	66	9.7
Other	2	0.3
Field of studying		
Medicine	249	36.5
Dentistry	10	1.5
Pharmacy	44	6.4
Nursing	256	37.5
Otherwise	124	18.2
Medical specialty		
Not a doctor	434	63.5
Internal medicine specialties	167	24.5
Surgeries	50	7.3
Pediatric medicine	12	1.8
Obstetrics and Gynecology	10	1.5
Anesthesia	10	1.5
During the corona lockdown, did you work at the hospital or a clinic, including private practice?		
No	67	9.8
Yes	616	90.2
How often did you have to work at the hospital or clinic (including private practice)?		
Never	31	4.5
Once a week	15	2.2
2-3 days a week	103	15.1
4-5 days a week	236	34.6
All weekdays including weekend	298	43.6
If you worked at the hospital or clinic, did other people, such as neighbors, relatives, or coworkers, know that you did?		
No	46	6.7
Yes	637	93.3
Were your neighbors, relatives, and coworkers aware of your contact with people infected with coronavirus?		
No	117	17.1
Yes	566	82.9
Did you have contact with people infected with coronavirus, including covid-19 patients?		
No	151	22.1
Yes	532	77.9
How extensive was your contact with people infected with covid-19?		
Only occasional contact for a few minutes with protective equipment	421	61.6
Close daily contact but with PPE	262	38.4
When the vaccine is available, N= 652		
I won't take it	260	38.1
I'll take it, but I’m worry	226	33.1
I'll take it, and I’m reassured	164	24.0
The vaccine will be dangerous	2	0.3

Most participants (90%) worked in a healthcare facility during the pandemic, with 78% working more than four days a week. About 43.6% of the respondents worked on weekends. Most of those around them (93.3%) were aware of their work during the pandemic, with 82.9% knowing of their direct contact with COVID-infected patients (Table 1).

The Specialty hospital had the most significant representation among the participants (25%), followed by the University of Jordan hospital (23.9%) and Istiklal hospital (11.4%). Most respondents (80%) were university graduates, and medical and nursing staff constituted 74% of the participants (Table 1).

This study showed that 260 respondents (38.1%) reported that they would not take the COVID-19 vaccine once it becomes available, while the rest expressed willingness to take it, regardless of any concerns they may have about its outcomes.

QoL

The results show that the minimum score for all items is 1, while the maximum score for all items is 5, indicating the lowest and highest possible levels of satisfaction for each factor among respondents as measured by the Likert scale for each question. The mean score for each item ranges from 3.34 to 3.99, with an overall mean QOL score of 3.71. The highest scores of respondents in health, overall QoL, and self-esteem were 3.99, 3.93, and 3.92, respectively, while the lowest was for the financial status domain. The standard deviation for each item ranges from 0.768 to 1.114, with an overall standard deviation of 0.6773 for the QOL score. These results suggest moderate to high levels of satisfaction with various aspects of daily living among respondents, with some variation in reported satisfaction levels as indicated by the range of scores and standard deviations (Table 2).

**Table 2:** EUROHIS-QOL 8-item and QoL among healthcare workers Quality of Life (QOL)

The EUROHIS-QOL 8-item index	N	Minimum	Maximum	Mean	Std. Deviation	Skewness	
How satisfied are you with your ability to perform your daily living activities	683	1	5	3.70	1.078	-.765	
How satisfied are you with your health	683	1	5	3.99	.945	-1.030	
How satisfied are you with yourself	683	1	5	3.92	.972	-.948	
How satisfied are you with your personal relationships	683	1	5	3.69	1.114	-.780	
How satisfied are you with the conditions of your living place	683	1	5	3.75	1.104	-.889	
How would you rate your quality of life	683	1	5	3.93	.768	-.975	
Do you have enough energy for everyday life	683	1	5	3.37	1.001	-.396	
Have you enough money to meet your needs	683	1	5	3.34	1.012	-.371	
QOL	683	1.25	5	3.71	.6773	-.538	

Depression-anxiety-stress in healthcare workers during the COVID-19 pandemic

Our study, which was conducted during the COVID-19 pandemic, revealed that more than half of healthcare workers in Jordan experienced some level of psychological distress during the pandemic, with depression being the most commonly reported (65%), followed by anxiety (61%) and stress (56%). Upon further analysis, we found that the severity of anxiety was predominantly extreme, affecting 25% of those who reported experiencing anxiety. At the same time, stress was mainly severe, affecting 18% of those who reported stress. In contrast, depression was primarily in the moderate range. The researchers could conclude that the COVID-19 pandemic might strongly correlate with the severity of and struggles with anxiety and stress among our healthcare workers (Table 3). 

**Table 3:** Frequency of DASS in healthcare workers during the COVID-19 pandemic DASS: Depression, Anxiety and Stress Scale.

DASS	Normal N (%)	Mild N (%)	Moderate N (%)	Severe N (%)	Extremely severe N (%)
Depression	242 (35%)	81 (12%)	162 (24%)	91 (13%)	107 (16%)
Anxiety	269 (39%)	50 (7%)	116 (17%)	74 (11%)	174 (25%)
Stress	299 (44%)	99 (14%)	107 (16%)	125 (18%)	53 (8%)

Interestingly, we have investigated this correlation by doing a chi-square test between which specialty has most of the stress related to the pandemic. We found that internal medicine specialties and nurses who were the front liners during the pandemic had the highest level of stress (extremely severe) during the pandemic - 8.4% and 7.6%, respectively, compared to the surgical specialties, which were only 4% with significant correlation (P value = 0.016). As expected, we also found that those who had more exposure to or contact with patients with COVID-19 (by asking them "did you have contact with people infected with coronavirus, including COVID-19 patients?") had higher levels of anxiety (63.5%) and stress (59.6%), as well as their extreme severity, is almost twice as much compared to non-exposed participants with significant correlation (P = 0.005, 0.008 respectively), as shown in Table 4.

**Table 4:** Degree of difference between DASS and medical specialty and contact with COVID patients DASS: Depression, Anxiety and Stress Scale.

Medical specialty	Normal N (%)	Mild stress N (%)	Moderate stress N (%)	Severe stress N (%)	Extremely severe stress N (%)	P-value
Internal medicine specialties, n= 167	84 (50.3%)	20 (12%)	18 (10.8%)	31 (18.6%)	14 (8.4)	0.016
Not physicians (including nurse, radiological staff), n= 434	183 (42.2%)	59 (13.6%)	80 (18.4%)	79 (18.2%)	33 (7.6)	
Surgical specialties, n= 50	24 (48%)	9 (18%)	5 (10%)	10 (20%)	2 (4%)	
Did you have contact with people infected with coronavirus including COVID-19 patients?						
Yes, n= 532	215 (40.4%)	76 (14.3%)	89 (16.7%)	106 (19.9%)	46 (8.6%)	0.008
No, n=151	84 (55.6%)	23 (15.2%)	18 (11.9%)	19 (12.6%	7 (4.6)	
Did you have contact with people infected with coronavirus including COVID-19 patients?						
	Normal N (%)	Mild Anxiety N (%)	Moderate Anxiety N (%)	Severe Anxiety N (%)	Extremely severe Anxiety N (%)	P-value
Yes, n= 532	194 (36.5%)	36 (6.8%)	90 (16.9%)	61 (11.5%)	151 (28.4%)	0.005
No, n= 151	75 (49.7%)	14 (9.3%)	26 (17.2%)	13 (8.6%)	23 (15.2%)	

Frequency of stigma among healthcare workers and its correlation

Table 5 displays the frequency distribution of stigma experience. The average participant count and percentage for each stigma category for each subscale and novel COVID-19 Stigma Scale among Healthcare Workers (COVISS-HCWs) were determined. Our findings showed that 3% of the participants had experienced stigmatization; 4% and 4.8% of the participating healthcare professionals reported feelings of harm, inferiority, and avoidance, respectively. 

**Table 5:** Frequency of stigma among healthcare workers

Stigma aspects, n= 683	No stigma, N (%)	Neutral, N (%)	Stigma present, N (%)
Social stigma	531 (78%)	135 (20%)	17 (3%)
Harmfulness and inferiority	536 (79%)	122 (18%)	25 (4%)
Avoidance Subscale	524 (76.7%)	126 (18.4%)	33(4.8%)

We utilized the chi-square test to assess the distribution of answers across demographic characteristics (Table 6). Furthermore, there was a direct response relationship between financial status and stigma experience (P = 0.003), with a higher percentage of low-income participants reporting stigma (8.6%) as opposed to 2.9%, 1.7% and 2.2% of participants with medium, sound, and excellent financial status, respectively. No discernible difference was found between medical specialty, educational level, and stigmatization frequency.

**Table 6:** Degree of difference between study variables and stigma

Variables		Social stigma			
		No stigma	Neutral	Stigma present	P-value
		Percent %			
Financial status	Low	25 (71.4%)	7 (20%)	3 (8.6%)	0.003
	Medium	145 (70%)	56 (27.1%)	6 (2.9%)	
	Good	283 (80.4%)	63 (17.9%)	6 (1.7%)	
	Excellent	78 (87.6%)	9 (10.1%)	2 (2.2%)	
Did you have contact with people infected with coronavirus including covid-19 patients?	No	134 (88.7%)	16 (10.6%)	1 (0.7%)	0.001
	Yes	397 (74.6%)	119 (22.4%)	16 (3%)	
How extensive was your contact with people infected with covid-19?	Only occasional contact for a few minutes with protective equipment	348 (82.7%)	69 (16.4%)	4 (1%)	0.000
	Close daily contact but with protective equipment	183 (69.8%)	66 (25.2%)	13 (5%)	
Age	18-30	363 (79.1%)	85 (18.5%)	11 (2.4%)	0.489
	31-50	146 (73.7%)	46 (23.2%)	6 (3%)	
	>=50	22 (84.6%)	4 (15.4%)	0 (0%)	
Gender	Male	208 (75.6%)	60 (21.8%)	7 (2.5%)	0.535
	Female	323 (79.2%)	75 (18.4%)	10 (2.5%)	
Place of residence	Capital	425 (80%)	96 (18.1%)	10 (1.9%)	0.141
	Northland	16 (61.5%)	9 (34.6%)	1 (3.8%)	
	Heartland	87 (71.3%)	29 (23.8%)	6 (4.9%)	
	Southland	3 (75%)	1 (25%)	0 (0%)	
Living condition	University housing	13 (76.5%)	4 (23.5%)	0 (0%)	0.651
	House	410 (78.7%)	97 (18.6%)	14 (2.7%)	
	Flat	108 (74.5%)	34 (23.4%)	3 (2.1%)	
How many people live in your household?	Alone	29 (78.4%)	8 (21.6%)	0 (0%)	0.264
	1-2	75 (70.8%)	27 (25.5%)	4 (3.8%)	
	3-4	196 (81.7%)	37 (15.4%)	7 (2.9%)	
	≥5	231 (77%)	63 (21%)	6 (2%)	
Have you traveled during the covid-19 period ?	No	508 (77.9%)	127 (19.5%)	17 (2.6%)	0.482
	Yes	23 (74.2%)	8 (25.8%)	0 (0%)	
Educational level	Doctorate	18 (85.7%)	3 (14.3%)	0 (0%)	0.7688
	Master degree	38 (82.6%)	8 (17.4%)	0 (0%)	
	University degree	425 (77.6%)	108 (19.7%)	15 (2.7%)	
	Institute degree	49 (74.2%)	15 (22.7%)	2 (3%)	
	Otherwise	1 (50%)	1 (50%)	0 (0%)	
Field of studying	Medicine	192 (77.1%)	55 (22.1%)	2 (0.8%)	0.051
	Dentistry	9 (90%)	0 (0%)	1 (10%)	
	Pharmacy	37 (84.1%)	6 (13.6%)	1 (2.3%)	
	Nursing	189 (73.8%)	56 (21.9%)	11 (4.3%)	
	Otherwise	104 (83.9%)	18 (14.5%)	2 (1.6%)	
Medical specialty	Not a doctor	339 (78.1%)	80 (18.4%)	15 (3.5%)	0.539
	Internal medicine specialties	133 (79.6%)	33 (19.8%)	1 (0.6%)	
	Surgeries	36 (72%)	13 (26%)	1 (2%)	
	Pediatric medicine	8 (66.7%)	4 (33.3%)	0 (0%)	
	Obstetrics and gynecology	7 (70%)	3 (30%)	0 (0%)	
	Anesthesia	8 (80%)	2 (20%)	0 (0%)	
During the corona lockdown, did you work at the hospital or a clinic including private practice?	No	52 (77.6%)	13 (19.4%)	2 (3%)	0.961
	Yes	479 (77.8%)	122 (19.8%)	15 (2.4%)	
How often did you have to work at the hospital or clinic (including private practice)?	Never	24 (77.4%)	5 (16.1%)	2 (6.5%)	0.646
	Once a week	10 (66.7%)	5 (33.3%)	0 (0%)	
	2-3 days a week	84 (81.6%)	18 (17.5%)	1 (1%)	
	4-5 days a week	182 (77.1%)	47 (19.9%)	7 (3%)	
	All week days including weekend	231 (77.5%)	60 (20.1%)	7 (2.3%)	
If you worked at the hospital or clinic did other people such as neighbors, relatives, or coworkers know that you did?	No	35 (76.1%)	10 (21.7%)	1 (2.2%)	0.935
	Yes	496 (77.9%)	125 (19.6%)	16 (2.5%)	
Were your neighbors, relatives, and coworkers aware of your contact with people infected with coronavirus?	No	96 (82.1%)	20 (17.1%)	1 (0.9%)	0.307
	Yes	435 (76.9%)	115 (20.3%)	16 (2.8%)	
When vaccine is available	I won't take it	197 (75.8%)	52 (20%)	11 (4.2%)	0.313
	I'll take it but I'm worry	182 (80.5%)	41 (18.1%)	3 (1.3%)	
	I'll take it and I'm reassured	129 (78.7%)	32 (19.5%)	3 (1.8%)	
	vaccine will be dangerous	1 (50%)	1 (50%)	0 (0%)	

Most participants (93.3%) had neighbors, family members, or coworkers who were aware that they worked in hospitals or clinics or had contact with people who were infected with the coronavirus (82.9%); however, our findings did not show a direct relationship with stigmatization. Regardless of differences in our respondents' characteristics, such as age, gender, location of residence, educational levels, area of study, kind of specialty, location of work, and the number of shifts per week, there was no significant influence on stigma. Furthermore, there is no association between their attitudes regarding acquiring a COVID-19 vaccination once it is available.

Additionally, the majority of participants (77.9%) had sporadic or frequent interaction with COVID-19 patients; stigmatization was strongly linked with the amount of contact with infected individuals (P = <0.001). In comparison 1% of those with infrequent interaction felt stigmatized, compared to 5% of those in close daily contact. In contrast, just 0.7% of those without interaction with COVID-19 patients felt stigmatized (Table 6).

Stigmatization correlates with psychological discomfort (depression-anxiety-stress) among healthcare workers

Table 7 reveals a significant relationship exists (P = < 0.001) between the feeling of stigmatization and the severity of psychological discomfort in depression, anxiety, and stress. Furthermore, stigmatization was experienced by 9.3%, 7.5%, and 3.8% of participants with highly severe depression, anxiety, and stress, respectively, compared to 0%, 0.4%, and 1% of people without significant psychological distress. These findings imply that persons who experience psychological pain of any intensity are more likely to experience stigmatization.

**Table 7:** Stigmatization correlates with psychological discomfort (depression-anxiety-stress) among healthcare workers

DASS			Social Stigma			P-value
			No stigma	Neutral	Stigma present	
Depression	Normal	F	220	22	0	<0.001
		%	90.9%	9.1%	0.0%	
	Mild	F	63	18	0	
		%	77.8%	22.2%	0.0%	
	Moderate	F	118	38	6	
		%	72.8%	23.5%	3.7%	
	Severe	F	63	27	1	
		%	69.2%	29.7%	1.1%	
	Extremely Severe	F	67	30	10	
		%	62.6%	28.0%	9.3%	
Anxiety	Normal	F	238	30	1	<0.001
		%	88.5%	11.2%	0.4%	
	Mild	F	40	10	0	
		%	80.0%	20.0%	0.0%	
	Moderate	F	87	26	3	
		%	75.0%	22.4%	2.6%	
	Severe	F	61	13	0	
		%	82.4%	17.6%	0.0%	
	Extremely Severe	F	105	56	13	
		%	60.3%	32.2%	7.5%	
Stress	Normal	F	264	32	3	<0.001
		%	88.3%	10.7%	1.0%	
	Mild	F	72	25	2	
		%	72.7%	25.3%	2.0%	
	Moderate	F	78	28	1	
		%	72.9%	26.2%	0.9%	
	Severe	F	86	30	9	
		%	68.8%	24.0%	7.2%	
	Extremely Severe	F	31	20	2	
		%	58.5%	37.7%	3.8%	

## Discussion

Stigma is defined as a substantial lack of respect for a person or a group of people or a wrong opinion of them because they have done something society does not approve of [17].

Many diseases and situations can cause some form of stigma toward a person or group of people who may carry some distinctions, such as when a person is considered to be the cause of a disease, to be contagious, or when a condition has some visible external signs on the patient's body. During the COVID-19 outbreak, many healthcare workers have been avoided by other people to protect themselves.

Yuan et al. conducted a study which revealed that there is a significant correlation between one's financial situation and the likelihood of being stigmatized, with 37% of individuals affected. However, in our study, low economic status is correlated with stigma (8.6%). As a result, low-income countries may have more significant psychological burdens on the population and healthcare workers because of stress [18].

Healthcare providers who worked with people infected with coronavirus are more stigmatized than those who did not work with COVID-19 patients during the pandemic. Furthermore, the current study shows that healthcare providers with close daily contact with COVID-19 patients were more stigmatized than others with only a few minutes of contact, 5%, and 1%, respectively. This finding aligns with previous studies, which found that healthcare workers who deal with COVID-19 patients are stigmatized 3.5 times quite as much [19].

Literature reports stigma to individuals who live in Northeast India during the pandemic due to facial similarities to people of Chinese descent. Also, one study from Iran found a low degree of stigmatization among those who live in the capital city, which might be attributed to the city's significant social desirability impact. In our investigation, there was no link [20,21].

Forty-six studies show that stigmatization arises because of work-related COVID-19 exposure. Previous studies found that healthcare workers exposed to COVID-19 faced stigma and repudiation in their societies compared to those who were not. Moreover, Yufika et al. showed that daily exposure of healthcare workers to COVID-19 eventually leads to stigma. Accordingly, stigma arises as the workload increases. While in our study, there was no relation between how many days they worked and being stigmatized [19,22,23].

Unfortunately, many healthcare workers have felt stigmatized during the pandemic, and many have had worries about transmitting COVID-19 infection to their beloved ones, all of which have affected the QoL of the healthcare workers [20,14,24].

Using the novel COVISS-HCWs, it was found that the prevalence of stigmatization during the COVID-19 pandemic among 683 healthcare workers in Jordan was estimated to be around 3% (p=0.043).

Comparably, in a recent study by Al Houri et al., stigma was reported to be around 9.86% among participants from significant hospitals in Syria [14]. Stigma was linked to lower socioeconomic status, younger age, and higher intensity of contact with COVID-19 patients. Internationally, few other studies have used other validated or non-validated stigma tools, and unfortunately, stigma was detected in multiple countries. In Egypt, a study by Osman et al. found that around 40% of healthcare workers who participated in their research have suffered from stigma. They have explained this high percentage for many reasons, one of which is that some patients might hide their contact history with other COVID-19 patients because of the COVID-19 stigma [22].

Yufika et al. surveyed around 288 healthcare workers in Indonesia, and approximately 21.9% of the respondents had a stigma due to COVID-19. They have linked this stigma to not being a doctor, having participated in COVID-19 training, working at a non-private hospital, or working in a hospital without COVID-19 triage protocols [25]. These wide ranges of numbers can be due to using non-validated stigma tools, poor socioeconomic and financial status, and more contact with COVID-19 patients with less personal protective equipment (PPE) [14,26].

Unfortunately, many healthcare workers may feel stigmatized by society only because they care for COVID-19 patients. However, being infected with COVID-19 is much more stigmatizing [24]. The Al Houri et al. study found that SARS-Cov-2 vaccine attitudes were strongly and significantly correlated with feeling stigmatized; the more stigma they experienced, the more motivation they had to take the vaccine in order to get more protection against the COVID-19 infection [14].

Healthcare workers are more prone to being infected with COVID-19 than the general population [27]. In our study, the majority (61.9%) of the respondents declared they would take the vaccine once it became available. The rest of the responses indicate that the respondents will not take the vaccine. Thus, governments should establish strict vaccination regulations or policies among healthcare workers.

Pandemic outbreaks, such as those caused by Ebola and SARS, have produced uncertainty and panic in the past. These unfavorable psychological reactions are linked to emotions of stress, worry, and sadness, all of which can lead to mental diseases [28,29]. Global COVID-19 pandemic research negatively affects previously healthy individuals and those with mental health concerns [30,31]. A systematic review demonstrated that at least one in five healthcare workers had reported symptoms of depression and anxiety during the COVID-19 pandemic [32].

The current study found that more than half of the healthcare workers (who cared for patients during the COVID-19 pandemic) had significant levels of stress, anxiety, and depression. The stress and anxiety levels varied depending on the specialization of the healthcare workers and the quantity of exposure to COVID-19 patients. Meanwhile, the severity of depression varied depending on the type of patient.

A range of studies was conducted to analyze the impact of COVID-19 on various sectors and thematic areas in Jordan. One of the studies, a cross-sectional survey designed by Alhalaiqa et al. using 225 healthcare workers, shows almost similar results for depression, anxiety, and stress prevalence in Jordan [33].

Frontline healthcare personnel are more likely to have poor mental health outcomes and exhibit more depressive and anxious symptoms. They experience an overwhelming worry of contracting the illness and infecting their loved ones. Similar concerns have been expressed by frontline healthcare professionals directly involved in diagnosing, treating, and caring for COVID-19 patients. They are at risk of experiencing psychological distress and poor mental health [34]. This can be supported by comparing healthcare workers' mental health to the general population. In Jordan, a study by Abuhammad et al., with a similar measured scale to the current research, has detected that the general population's stress score was 13.2, and depression 11.95. Anxiety was 7.6, which is much lower than the present study results. Also, for those with a moderate-high probability of infection with COVID-19, their DASS scores showed significantly higher scores but still lesser than that of healthcare workers (stress 14.8, anxiety 8.77, and depression 13.33) [35]. In another study that supports the vulnerability of healthcare workers toward mental health distress compared to the general population, Naser et al. studied 2,961 individual participants in Jordan (general population=1,798, healthcare workers=1,163). Depression was most prevalent in healthcare workers (n=247, 21.2%) while in the general population (n=284, 15.8%). Similarly, anxiety was most prevalent across by healthcare workers (n=131, 11.3%) while general population (n=158, 8.8%) [36].

Indeed, it is critical to assess risk factors for psychological issues among healthcare workers, such as demographics and social features, to implement preventative or protective interventions and programs in practice settings. Several factors studied in the literature appear to operate as defensive mechanisms against mental health diseases, most notably perceived QoL [37,38]. A recent study in Brazil studied many protective factors for depression, including resilience, spirituality, social support, QoL, and other variables. Their results showed that QoL is the most potent protective factor against depression [39].

Measuring QoL is a good technique for assessing health status in populations from various occupational groups, including healthcare providers. This is the first study to evaluate Jordanian healthcare workers' QoL during COVID-19 using the EUROHIS-QOL scale.

Given the large number of deaths among healthcare workers, the COVID-19 pandemic has been believed to impact healthcare workers' QoL substantially. Furthermore, hospital environments are characterized by high work-related stress, a well-known risk factor for poor QoL [40]. The current study respondents' QoL during COVID-19 in Jordan is rated relatively low compared to the global adult population. As no other studies examining QoL using a similar scale for the general population in Jordan are known, we found two recent studies that used the same EUROHIS-QOL scale to measure QoL among the adult population in Germany and Saudi Arabia during the COVID-19 pandemic. These studies reported higher scores of 3.95 and 3.91, respectively, compared to the healthcare workers in Jordan who were the focus of the current study [41,42].

Another cross-sectional study conducted in Jordan by Almhdawi et al. used a different scale to measure the QoL among physicians during the COVID-19 pandemic. The 12-item Short Form health survey (SF-12) was used to assess both the mental and physical components of QoL. The study found that the QoL of Jordanian physicians was relatively low, which is consistent with our findings [43]. In addition to, a large multinational study on 19 Arab countries two years after the COVID-19 pandemic concluded that a significant proportion of Arab healthcare workers had an overall poor QoL; the mean scores of general health and general QoL were 3.7 ± 1.0 and 3.7 ± 0.9, respectively [44].

Ensuring that healthcare workers have an appropriate level of QoL is crucial since they are an essential component of the healthcare system framework. Their well-being is necessary for improved patient care and, as a result, safer results. Regretfully, most Arab healthcare workers tested had low QoL scores across the board. This is a concerning result that should be studied further.

Moreover, hospital administrators in practice must consider these risk factors when devising an effective intervention to address the requirements of healthcare workers to preserve their mental health. Before caring for COVID-19 patients, mental health programs must focus on intervention and preparing healthcare workers in clinical settings. Also, they must give mental health training to their healthcare workers to manage their stress and their patients' stress. All of that is needed to maintain our front liners to continue with their work and maintain optimum patient care.

The limitation of this study includes that self-reported data may not always accurately reflect the experiences of healthcare workers. However, it can still be a valuable tool for understanding the experiences and perspectives of healthcare workers. To address this, further research with methods of data collection, such as combining self-reported data with observations or objective measures, is required to obtain a more comprehensive understanding of the impact of COVID-19 on healthcare workers' stigma, stress, and QoL.

## Conclusions

Healthcare workers’ mental well-being has been negatively affected by the COVID-19 pandemic, resulting in depression, anxiety, and stress. Widespread mental surveillance for healthcare workers is crucial to protect healthcare workers from psychological issues and to improve the healthcare service provided to patients. Stigma among healthcare workers can be a significant factor that may increase depression, anxiety, and stress.
